# Short-term follow-up of embolization of hip synovitis

**DOI:** 10.1186/s42155-020-00126-1

**Published:** 2020-07-12

**Authors:** Mateus Picada Correa, Renan Camargo Puton, Jaber Nashat Saleh, Rafael Stevan Noel, Luis Henrique Penteado da Silva, Daniela Medeiros de Castro, Julio Cesar de Mello Bajesrki

**Affiliations:** 1 Universidade de Passo Fundo, Universidade Meridional, Instituto Vascular de Passo Fundo (Invasc), Rua Capitão Araújo, 297/1210, Passo Fundo, RS 99010-200 Brazil; 2 Invasc, Rua Capitão Araújo, 297/1210, Passo Fundo, RS 99010-200 Brazil; 3 Universidade de Passo Fundo (UPF), Invasc, Rua Capitão Araújo, 297/1210, Passo Fundo, RS 99010-200 Brazil; 4 Instituto de Ortopedia e Traumatologia (IOT), R. Uruguai, 2050, Passo Fundo, RS 9010-112 Brazil; 5 Clínica Kozma - Diagnóstico por Imagem, R. Teixeira Soares, 793, Passo Fundo, RS 99010-080 Brazil

**Keywords:** Embolization, Embolotherapy, Inflammatory embolization, Pain management, Synovitis, Hip, Inflammation, Ostheoartritis

## Abstract

**Background:**

Osteoarthritis (OA) is the most frequent joint disease, affecting 10% of men and 18% of women older than 60 years worldwide. Traditionally, treatment is based in pain management with joint replacement of end-stage disease. In this setting, transcatheter embolization has emerged as an alternative in reduction of pain in patients with OA.

**Case presentation:**

A 77 years-old female presenting with two previous deep vein thrombosis and 10 years of hip pain. Magnetic resonance demonstrated a focal area of enhanced pericapsular signal near the superolateral margin of the acetabulum. Embolization of branches of the ascending branch of the lateral femoral circumflex artery was performed, with decrease of the pain and improvement in the image and her walking ability in a four-month follow-up.

**Conclusion:**

This case report have shown that embolization of hip synovitis is feasible with early clinical success, in tune with the findings of genicular and shoulder embolization. Studies with more patients and long-term results are necessary to corroborate this finding.

**Level of evidence:**

4

## Background

Osteoarthritis (OA) is the most frequent joint disease, affecting 10% of men and 18% of women older than 60 years worldwide (Glyn-Jones et al. 2015). The disease may cause debilitating pain and socioeconomic spent.

Traditionally, treatment was based in pain management with joint replacement in the end-stage disease. There are reports of minimally invasive treatments of knee pain, such as radiofrequency and shock wave therapy. However, these treatments have mixed results. Transcatheter embolization has emerged as an alternative in reduction of pain in patients with OA (Okuno et al. 2015). This paper presents the first report of a successful hip synovitis embolization.

## Case presentation

**Fig. 1:**
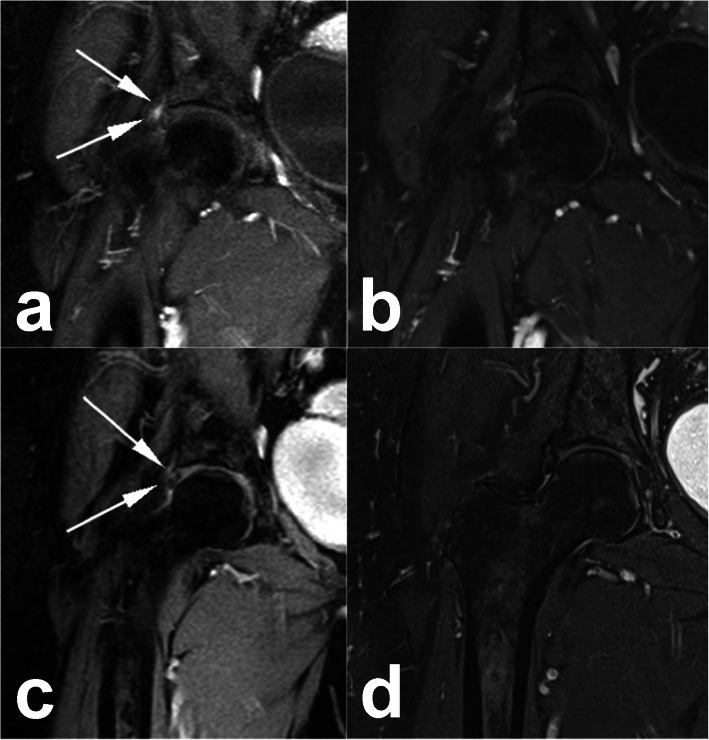
Coronal Magnetic Resonance image of the right hip joint. **a** and **b** pre and post embolization DPW-weighted image with adipose tissue saturation. **c** and **d** pre and post embolization Post-contrast T1W-weighted image with tissue saturation. In (**a** and **c**) note a degeneration in the superolateral segment of the acetabular labrum with mild pericapsular edema and post-contrast enhancement (arrows). In the four- month post-procedure image, the pericapsular edema was almost completely resolved (**b**), and a partial resolution of post-pericapsular contrast enhancement (**d**)

A 77 years-old female presenting with a history of 10 years of pain in her right hip worsened during movement. She had a past history of right knee arthoplasty and two episodes of deep vein thrombosis (DVT). She was referred to our clinic by the orthopedic team in use of rivaroxaban 20 mg once a day and physical therapy twice a week, with no improvement in her hip pain. Her visual analogue scale (VAS) of pain was 8 in 10. Magnetic resonance imaging (MRI) demonstrated a focal area of enhanced pericapsular signal near the superolateral margin of the acetabulum. This was the area of pain in the physical exam (Fig. 1a and c).

**Fig. 2:**
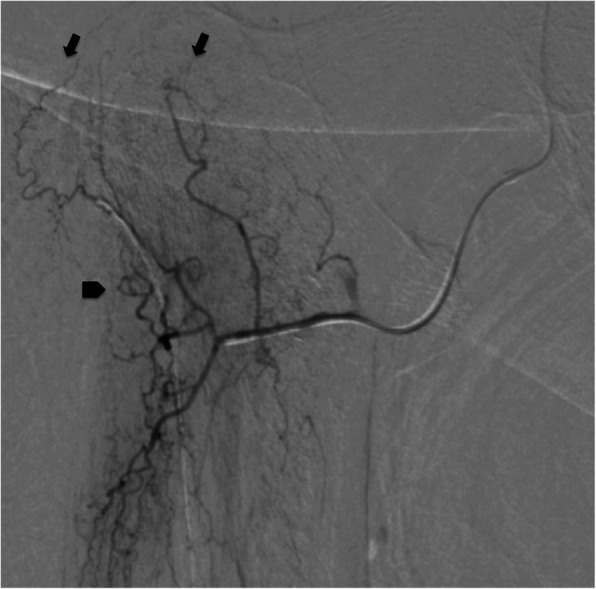
Super selective angiography of the ascending branch of the lateral femoral circumflex artery with a 2.4Fr micro catheter. The “inflammatory blush” was not found, however, small corkscrew arteries ascending to he point of hipersignal in the CT were found (arrows). There were a few more corkscrew-shaped arteries in a point of pain during the procedure (arrowhead), which were also occluded

**Fig. 3:**
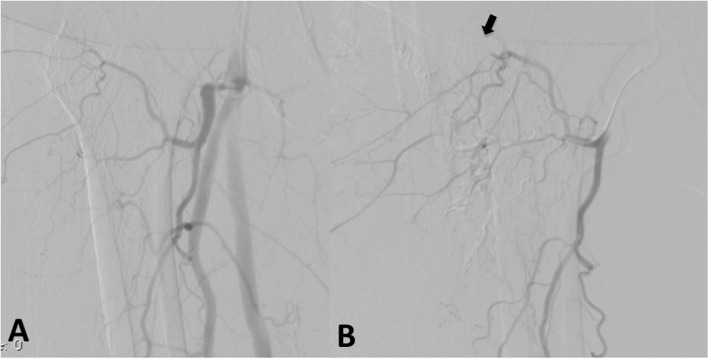
Pre (**a**) and post (**b**) angiogram of the profunda femoris demonstrating the area of stasis at the more superior branch ascending branch of the lateral femoral circumflex artery (arrow)

After a discussion of options, she agreed with inflammatory embolization. Due to her small stature, the procedure was started using radial access, which was aborted due to the lack of length of the devices. A contralateral femoral access was used for access of the right profunda femoris artery using a JIM 5Fr catheter. A 2,4Fr Progreat (Terumo, Japan) microcatheter was inserted coaxially and access to superior branches of the ascending branch lateral femoral circumflex artery was achieved. Blush of the inflammatory area was not found, only small corckscrew branches on the areas that the patient referred pain in the injection. It was injected 0.2 cc of 100-300 μm of BeadBlok microspheres (BTG; Farnhan, UK) in each vessel, until stasis was found (Figs. 2 and 3). The patient reported a VAS of 6 by the end of the procedure, reducing to zero in the next day. Additionally, her hip mobility was improved. At the four-month follow-up visit, patient was still with VAS of zero and was not using her walking stick. The 4-month control MRI demonstrated an almost-complete resolution in the pericapsular edema and a partial improvement in the post-contrast enhancement (Fig. 1b and d).

## Discussion

OA is a multifactorial disease classified as primary or secondary based on the recognized causative factors (Martel-Pelletier et al. 2016). Age and obesity are considered the most prominent, however, trauma, surgery or congenital abnormalities in joints are few examples of risk factors.

Okuno et al. postulated embolization of the inflammatory regions of joints causing pain (Okuno et al. 2015). The technique was already been performed in the treatment of plantar fasciitis, and to treat synovitis of knee and upper limb joints with excellent short and middle-term results (Okuno et al. 2015; Okuno et al. 2017a; Hwang et al. 2018; Okuno et al. 2017b).

The rationale of the technique comprises in understanding that the pathological mechanisms of OA involves pro-inflammatory leukins, such as IL-1β, IL-6 and IL-8 and tumor necrosis factor (TNF) α. These inflammatory mediators, in addition to mechanical and oxidative stress, cooperate to compromise the function and viability of chondrocytes, reprogramming them to undergo an hypertrophic differentiation, making them more sensitive to the effects of pro-inflammatory and pro-catabolic mediators (Mobasheri and Batt 2016).

This pro-inflammatory state of the joint activates innumerous receptors, and the vascular endothelial growth factor (VEGF) represents the major rate-limiting step during of the process from the angiogenic process. In resume, chronic inflammation and angiogenesis are reciprocal cause and effect factors that aggravate and intensify each other (Costa et al. 2007). The vicious circle is deteriorated by the genesis of abnormal enervation. Ashraf et al. found in postmortem knees with high tibiofemoral chondropathy an increased density of blood vessels near the fibrocartilage junction, in association with a great number of perivascular sensory nerves, postulating that this association is a possible mechanism of pain in OA (Ashraf et al. 2011).

To our knowledge, this is the first publication of hip inflammatory embolization. There are few technical points in this case that must be highlighted: first, it was not found the inflammatory “tumoral” blush seen in other publications. The embolization was performed based only in the branches that the patient referred pain during selective angiography and the identification of small corkscrew arteries at that area. This may be due to the small focal area of hipersignal found on MRI. Despite performing the procedure with technical success, it may not be recommend embolization without identification of the blush, since no-target embolization can happen, specially if superselective catheterization is not achieved. Second, despite there are reports of use of microspheres in this technique (Okuno et al. 2017a), there is a risk of hip osteonecrosis there is so far unknown and must be considered, particularly if non-target embolization of the proximal branches of the lateral femoral circumflex artery occur. Imipenen/cilastatin was not used in this first case due to the absent of the authorization of our institution for the arterial use of the drug. In the following cases (unpublished data) the authors have shifted to its use, since it was demonstrated by Woodhams et al. that this embolic agent may reduce the risk of ischemia (Woodhams et al. 2013). Third, the procedure was performed with local anesthesia. Collaboration of the patient, reporting her symptoms, was paramount for the technical success. Despite the small stature of our patients (149 cm) this was not possible to perform the procedure using radial access since a very distal super-selective catheterization is necessary. In this situation, contralateral approach is an option.

## Conclusion

This case report have shown that embolization of hip synovitis is feasible with early clinical success. The four months follow-up was reported since Okuno et al. in their first reports have also published data in this time frame (Hwang et al. 2018). Studies with more patients and long-term results are necessary to corroborate these findings.

## Data Availability

Not applicable.
